# Supplementary material for: Survival Differences by Comorbidity Burden among Patients with Stage I/II Non-Small-Cell Lung Cancer after Thoracoscopic Resection
Source: Cancers (Basel). 2023 Mar 30;15(7):2075. doi: 10.3390/cancers15072075 (PMC10093192; doi:10.3390/cancers15072075)

Supplementary Table S1. Sensitivity analysis for participants not receiving chemotherapy (N=52,083)

	**No. deaths/person-years**	**Mortality Rate (per 1,000 person-years) (95% CI)**	**Age-adjusted HR (95% CI)**	**aHR (95% CI) †**	**aHR (95% CI) §**
**CCI**					
**0**	6,335/99,920.61	63.4 (61.9-64.9)	1 [Reference]	1 [Reference]	1 [Reference]
**1**	5,404/61,968.89	87.2 (85.0-89.5)	1.37 (1.32-1.42)	1.29 (1.25-1.34)	1.26 (1.22-1.31)
**2+**	3,430/29,905.93	114.7 (111.1-118.4)	1.78 (1.71-1.86)	1.62 (1.55-1.69)	1.56 (1.49-1.62)
			p-trend < 0.01	p-trend < 0.01	p-trend < 0.01

Abbreviations: **aHR:** Adjusted hazard ratio; **CCI:** Charlson comorbidity index; **CI:** Confidence interval

† Adjusted for age, sex, race/ethnicity, education, income, insurance, and facility type (academic versus nonacademic)

§ Adjusted for all covariates included in first model, as well as stage at diagnosis, days from diagnosis to surgery and histological type

Supplementary Table S2. Sensitivity analysis incorporating different sets of covariates and indicator of exclusion due to missing data

	**Model 1 (N=66,508)**<br>**aHR (95% CI)**	**Model 2 (N=75,701)**<br>**aHR (95% CI)**	**Model 3 (N=63,061)**<br>**aHR (95% CI)**
**CCI**			
0	1 [Reference]	1 [Reference]	1 [Reference]
1	1.26 (1.22-1.30)	1.26 (1.22-1.30)	1.23 (1.19-1.27)
2+	1.56 (1.51-1.62)	1.57 (1.52-1.63)	1.55 (1.45-1.56)
	p-trend < 0.01	p-trend < 0.01	p-trend < 0.01
**Adjusted variables**	Age, sex, race/ethnicity, income, education, and indicator of exclusion	Age, sex, race/ethnicity, insurance, facility type, and indicator of exclusion	Age, sex, race/ethnicity, histological type, tumor stage, receipt of chemotherapy, days from diagnosis to surgery, and indicator of exclusion
**No. of additional cases in model**	4,748	13,941	1,301
**Ratio to study population‡**	1.08	1.23	1.02

‡The ratio is calculated as (No. patients included for sensitivity analysis/No. study population in main model [N=61,760])

Supplementary Table S3. Sensitivity analysis for 90-day mortality by CCI

CCI	No. deaths within 90 days after surgery	Proportion (%) of deaths and 95% CI
0 (N=31,623)	573	1.8 (1.7-2.0)
1 (N=19,640)	469	2.4 (2.2-2.6)
2+ (N=10,497)	337	3.2 (2.9-3.6)

Supplementary Table S4. Sensitivity analysis for participants who received sublobar resection (N=18,007)

	aHR (95% CI)			
**CCI**	**Wedge resection (N=13,951)**	**Segmental resection (N=3,611)**	**Other* (N=445)**	**p-interaction**
0	1 [Reference]	1 [Reference]	1 [Reference]	< 0.01
1	1.21 (1.13-1.29)	1.46 (1.27-1.68)	1.47 (1.05-2.05)	
2+	1.51 (1.41-1.63)	1.56 (1.33-1.84)	1.15 (0.79-1.69)	

*Includes unspecified excision, laser excision, and bronchial sleeve resection (aggregated due to low counts)

Model adjusted for age, sex, race/ethnicity, education, income, insurance, facility type (academic versus nonacademic), stage at diagnosis, histological type, days from diagnosis to surgery, and receipt of adjuvant chemotherapy

Supplementary Figure S1. Kaplan-Meier survival curve by CCI, including patients with missing values for relevant covariates.

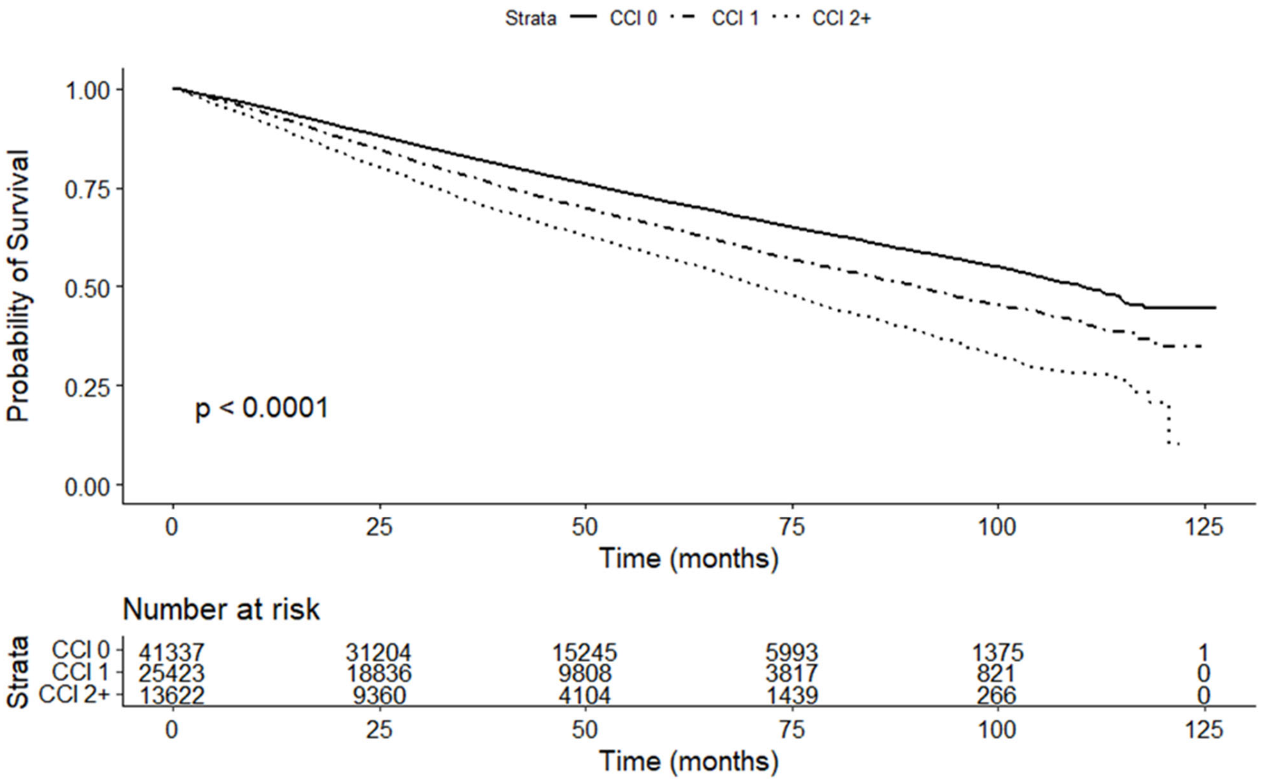

Supplement: Supplementary file 1 [file cancers-15-02075-s001.zip › cancers-2202032-supplementary.pdf]
